# Long-term disability in anxiety disorders

**DOI:** 10.1186/s12888-016-0946-y

**Published:** 2016-07-19

**Authors:** Sanne M. Hendriks, Jan Spijker, Carmilla M. M. Licht, Florian Hardeveld, Ron de Graaf, Neeltje M. Batelaan, Brenda W. J. H. Penninx, Aartjan T. F. Beekman

**Affiliations:** Department of Psychiatry, Pro Persona Mental Health Care, Zandstraat 54, Veenendaal, 3905 ED The Netherlands; Pro Persona Mental Health Care, Radboud University Nijmegen, Reinier Postlaan 6, Nijmegen, 6525 GC The Netherlands; Department of Psychiatry/EMGO Institute, VU University Medical Center, AJ Ernststraat 887, Amsterdam, 1081 HL The Netherlands; Pro Persona Mental Health Care, Willy Brandtlaan 20, Ede, 6717 RR The Netherlands; Netherlands Institute of Mental Health and Addiction, Da Costakade 45, Utrecht, 3521 VS The Netherlands

## Abstract

**Background:**

This longitudinal study aims to investigate differences in long-term disability between social anxiety disorder (SAD), panic disorder with agoraphobia (PDA), panic disorder without agoraphobia (PD), generalized anxiety disorder (GAD) and multiple anxiety disorders (multiple AD), focusing on the effects of different course trajectories (remission, recurrence and chronic course) and specific symptom dimensions (anxiety arousal and avoidance behaviour).

**Methods:**

Data were used from participants with no psychiatric diagnosis (healthy controls, *n* = 647) or with a current anxiety disorder (SAD, *n* = 191; PDA, *n* = 90; PD, *n* = 84; GAD, *n* = 110; multiple AD, *n* = 480). Severity of anxiety arousal and avoidance behaviour symptoms was measured using the Beck Anxiety Inventory and the Fear Questionnaire. The World Health Organization Disability Assessment Schedule II was used to measure disability.

**Results:**

Long-term disability was most prevalent in participants with SAD and multiple AD, and lowest in PDA and PD. GAD had an intermediate position. Anxiety arousal and avoidance behaviour were associated with more long-term disability in anxiety disorders than course trajectories.

**Conclusions:**

Various anxiety disorders have different disability levels over 4 years of time, therefore diagnostic distinction is important for treatment focus. Anxiety arousal and avoidance behaviour are major predictors for long-term disability in anxiety disorders.

## Background

Disability is often defined as ‘any restriction or lack of capacity to perform an activity in a manner or within a range considered normal for a human being’ [1]. Anxiety disorders are associated with severe disability [2–6] and the negative impact of anxiety is substantial [3, 7, 8]. Previous research showed that anxiety disorders differ in disability levels. Overall, multiple anxiety disorders (multiple AD, i.e. comorbidity with other anxiety disorders) are associated with more disability than pure anxiety disorders [9]. Furthermore, social anxiety disorder (SAD) and generalized anxiety disorder (GAD) are associated with higher disability levels compared to panic disorder, [3, 10–12] although other research points out otherwise [13–15]. However, it remains unclear whether contrasts in disability levels among anxiety disorders persist over a longer period.

More severe symptoms and more comorbidity are associated with a chronic course in anxiety disorders [16–20]. Therefore, we expect that long-term disability is more common in anxiety patients with a chronic course. However, some research gave indications that disability can still be present after remission of the anxiety disorder [21]. Because SAD and multiple AD are more strongly associated with a chronic course than other anxiety disorders [19, 22–24] we expect that long-term disability therefore is more prevalent in SAD and multiple AD.

Furthermore, previous research showed that symptom dimensions, like anxiety arousal and avoidance behaviour, can be strong predictors [19, 20, 25]. Hendriks et al. [19] showed that avoidance behaviour symptoms may be more important predictors than the symptoms of anxiety itself. This would lead to the hypothesis that long-term disability is more frequently seen in panic disorder with agoraphobia (PDA) and SAD compared to panic disorder without agoraphobia (PD) and GAD because of the high levels of avoidance behaviour in PDA and SAD.

This longitudinal study among a large cohort of participants with anxiety disorders investigates: 1) differences in long-term disability between participants with social anxiety disorder (SAD), panic disorder with agoraphobia (PDA), panic disorder without agoraphobia (PD), generalized anxiety disorder (GAD) and multiple anxiety disorders (multiple AD); 2) differences in long-term disability for different course trajectories; and 3) the role of anxiety arousal and avoidance behaviour in long-term disability.

## Methods

### Study sample

As reported before, [11, 19, 20]. The Netherlands Study of Depression and Anxiety (NESDA) is a naturalistic cohort study to examine the long-term course and consequences of anxiety and depressive disorders [26]. In short, a total of 2981 participants were included, aged 18 through 65 years. The research protocol was approved centrally by the ethical review board of VU University Medical Center. Subsequently it was approved by the local ethical review boards of Leiden University Medical Center and University Medical Center Groningen. The study was performed in accordance with the ethical standards of the Declaration of Helsinki. All participants provided written informed consent. Participants with a current or lifetime diagnosis of anxiety or depression, and healthy controls were included. Exclusion criteria were (1) a primary diagnosis of psychotic, obsessive compulsive, bipolar or severe addiction disorder, and (2) not being fluent in Dutch.

**Fig. 1:**
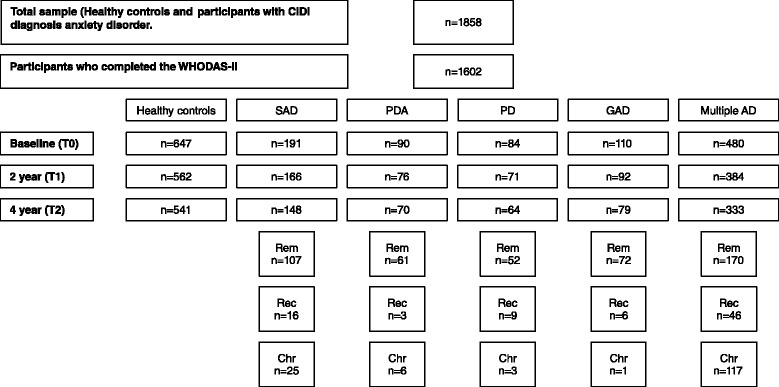
Flowchart of study sample

Participants with an anxiety disorder (SAD, PDA, PD, GAD and multiple AD) at baseline (6-month recency diagnosis) and healthy controls were included for this study (Fig. 1). A follow-up assessment was done two (T1) and 4 years (T2) after the baseline measurement. In total 1602 participants met these criteria at T0 (baseline); 191 participants (11.9 %) with pure SAD, 90 participants (5.6 %) with pure PDA, 84 participants (5.2 %) with pure PD, 110 participants (6.9 %) with pure GAD, 480 participants (30.0 %) with multiple AD and 647 healthy controls (40.4 %). 1351 participants (84.3 %) had a follow-up assessment at T1, and 1235 participants (77.1 %) at T2.

### Psychiatric status

As done before, [11, 19, 20] the Composite International Diagnostic Interview (CIDI version 2.1) was used to diagnose the presence of SAD, PDA, PD, GAD and multiple AD, a highly reliable and valid instrument for assessing anxiety disorders [27].

### Course trajectory

The clinical course trajectory categorized the participants into four groups: a) healthy controls (no history of anxiety disorder and no anxiety disorder at T0, T1 and T2), b) remission (remission of baseline anxiety disorder at T1 and/or T2 without recurrence), c) recurrence (remission of baseline anxiety disorder at T1 but with recurrence at T2), and d) chronic course (baseline anxiety disorder at T0, T1 and T2).

### Anxiety arousal and avoidance behaviour

As done before, [11, 19, 20] the Beck Anxiety Inventory (BAI) [28] and the Fear Questionnaire (Fear Q) [29] were used to measure baseline severity of anxiety arousal and avoidance behaviour symptoms, respectively. The BAI and the Fear Q are both widely used and have proven to be highly valid and reliable [30, 31]. The Cronbach’s α in this study for the BAI and the Fear Q were .94 and .89, respectively.

### Disability

To measure disability at baseline, after two and after 4 years, we used the total score of the World Health Organization Disability Assessment Schedule II.(WHODAS-II) [32]. The WHODAS-II provides a functioning profile for six activity domains (cognition, mobility, self-care, social interaction, life activities and participation). To measure general disability, domain scores were combined into a total score. The WHODAS-II shows good inter-item reliability with a Cronbach’s α of .96 for the total score. In Hendriks et al. [11] a cross sectional study was conducted to compare the different disability domains of the WHODAS-II between different anxiety disorders and healthy controls. The results showed that disability was generally highest in multiple anxiety disorder (e.g. mean disability in cognition = 33.7) and social anxiety disorder (mean = 32.7), followed by generalized anxiety disorder (mean = 27.2) and panic disorder with agoraphobia (mean = 26.3), and lowest in panic disorder without agoraphobia (mean = 22.1). This pattern was consistently present across different disability domains, therefore for this study we choose to use only the total score of the WHODAS-II.

### Covariates

As done before, [11] covariates at baseline were set: age, gender, years of education attained, partner status, number of somatic illnesses and comorbid depressive disorder. Previous studies showed that these covariates are associated with both anxiety and disability [33, 34].

### Statistical analyses

SPSS Version 20.0 (SPSS Inc, Chicago, Illinois) was used for the statistical analyses. We used chi-square statistics for categorical and analyses of variance for continuous variables to compare sociodemographic, clinical psychiatric characteristics and long-term disability between healthy controls and participants with anxiety disorders. Linear mixed models (LMM) [35] were used to analyse the relationship between 1) baseline psychiatric status, 2) course trajectories and 3) symptom dimensions with the outcome long-term disability.

## Results

Table 1 presents the demographic and clinical characteristics of the total sample. A chronic course was most prevalent in the multiple AD group (35.1 %). After 4 years 64.9 % of the participants with SAD, 68.6 % of PDA, 65.6 % of PD, 79.7 % of GAD and 51.1 % of multiple AD were without an anxiety disorder (*X*^2^ = 355.5, df = 25, *p* < .001). Anxiety arousal and avoidance behaviour symptoms were highest in the multiple AD group compared to other groups (BAI F = 286.2, df = 5, *p* < .001; Fear Q F = 205.12, df = 5, *p* < .001).

**Table 1:** Demographic and clinical characteristics of the total sample (healthy controls and participants with different anxiety disorders, *n* = 1602)

	Healthy controls	SAD	PDA	PD	GAD	Multiple AD	*p* ^a^
	*n* = 647	*n* = 191	*n* = 90	*n* = 84	*n* = 110	*n* = 480	
Sociodemographics							
Age (mean, SD)	41.1 (14.6)	37.3 (11.9)	40.1 (12.5)	38.3 (11.1)	37.4 (12.0)	40.8 (11.7)	.001
Sex (% female)	61.7	64.9	74.4	59.5	69.1	68.5	.05
Education (mean, SD)	12.8 (3.2)	12.6 (3.2)	12.1 (3.4)	12.4 (3.1)	12.4 (3.1)	11.3 (3.3)	<.001
Partner status (% yes)	75.0	56.5	70.0	73.8	70.0	63.8	<.001
Clinical psychiatric characteristics							
Number of somatic illnesses (mean, SD)	0.7 (1.0)	0.8 (0.9)	0.9 (1.0)	0.7 (0.8)	0.7 (0.9)	1.1 (1.2)	<.001
Comorbid depressive disorder (%)	0	41.9	38.7	40.5	62.7	72.5	<.001
Psychiatric course trajectories (%)							<.001
Remission	-	72.3	87.1	81.2	91.1	51.1	
Recurrence	-	10.8	4.3	14.1	7.6	13.8	
Chronic course	-	16.9	8.6	4.7	1.3	35.1	
Psychiatric status after 4 years (%)							<.001
No anxiety disorder	-	64.9	68.6	65.6	79.7	51.1	
Social anxiety disorder	-	20.9	2.9	3.1	7.6	16.8	
Panic disorder with agoraphobia	-	0	8.6	4.7	0	6.3	
Panic disorder without agoraphobia	-	2.0	8.6	12.5	3.8	6.3	
Generalized anxiety disorder	-	4.1	5.7	1.6	8.9	7.2	
Multiple anxiety disorder	-	8.1	5.7	12.5	0	12.3	
Symptom dimensions							
Anxiety arousal (BAI, mean, SD)	4.1 (4.9)	13.9 (9.1)	19.1 (11.6)	14.2 (8.9)	14.6 (8.2)	23.1 (11.1)	<.001
Avoidance behaviour (Fear Q, mean, SD)	12.1 (12.2)	34.4 (18.8)	32.1 (19.0)	22.8 (18.1)	25.0 (16.3)	43.7 (20.6)	<.001

^a^ *p*-value based on chi-square statistics for categorical variables and analyses of variances for continuous variables

**Fig. 2:**
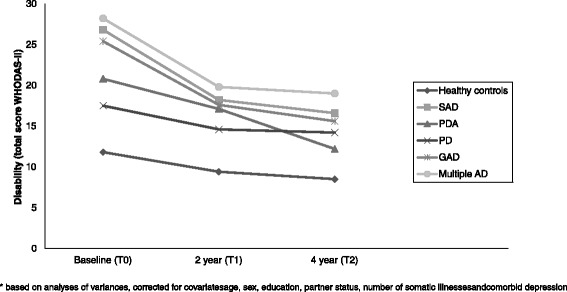
Disability (total score WHODAS-II) over 4 years of time for baseline psychiatric status (*n* = 1235) *

Figure 2 shows 4-year disability patterns between participants with SAD, PDA, PD, GAD, multiple AD and healthy controls (*n* = 1235) at each time point. Healthy controls experienced less disability compared to participants with any anxiety disorder and this differed significantly at baseline (all *p*-values < .001), 2-year follow-up (all *p*-values < .05) and 4-year follow-up (all *p*-values < .04). Generally, SAD and multiple AD participants experienced most disability. Disability was lowest in PDA and PD, and GAD had an intermediate position. At baseline, participants with SAD, GAD and multiple AD experienced more disability than participants with PDA and PD (all *p*-values < .03). Participants with multiple AD had more disability than participants with PD at 2 years (T1) (*p* = .01). At 4 years (T2), participants with SAD showed more disability than participants with PDA (*p* = .02), and participants with multiple AD showed more disability than participants with PD (*p* = .01) and PDA (*p* = .02).

Table 2 shows LMM analyses for long-term disability. Univariable analyses indicate that having an anxiety disorder at baseline predicts long-term disability. SAD x time, PDA x time, GAD x time and multiple AD x time showed significant negative associations. This indicates that during follow-up the impact of the disorders on long-term disability became smaller. Course trajectories (remission, recurrence and chronic course) and symptom dimensions (anxiety arousal and avoidance behaviour) were also associated with long-term disability. No associations were found for symptom dimensions x time, suggesting that associations did not significantly change over time.

**Table 2:** Linear mixed model analyses for long-term disability (total score WHODAS-II)^a^

	Disability							
	Univariable		Model 1		Model 2		Model 3	
	ß (SE)	*p*	ß (SE)	*p*	ß (SE)	*p*	ß (SE)	*p*
Baseline psychiatric status								
Healthy controls	Ref		Ref		Ref		Ref	
SAD	15.28 (1.55)	<.001	14.26 (1.74)	<.001	5.09 (1.42)	<.001	4.38 (1.55)	<.001
SAD x time	-6.48 (0.61)	<.001	-6.40 (0.61)	<.001	-2.52 (0.57)	.01	-2.56 (0.57)	.01
PDA	9.31 (2.09)	<.001	9.54 (2.24)	<.001	-0.46 (1.88)	.65	-0.65 (1.98)	.52
PDA x time	-4.18 (0.85)	<.001	-4.04 (0.85)	<.001	-0.07 (0.77)	.94	-0.11 (0.77)	.92
PD	5.96 (2.15)	<.001	6.40 (2.29)	<.001	0.46 (1.84)	.65	0.23 (1.94)	.82
PD x time	-1.43 (0.88)	.15	-1.29 (0.88)	.20	-0.07 (0.77)	.95	-0.11 (0.77)	.92
GAD	13.01 (1.93)	<.001	13.19 (2.07)	<.001	6.62 (1.68)	<.001	6.07 (1.77)	<.001
GAD x time	-5.80 (0.79)	<.001	-5.58 (0.80)	<.001	-3.52 (0.70)	<.001	-3.55 (0.70)	<.001
Multiple AD	20.93 (1.57)	<.001	18.21 (1.58)	<.001	3.58 (1.46)	<.001	3.00 (1.58)	.003
Multiple AD x time	-7.61 (0.54)	<.001	-7.54 (0.54)	<.001	-1.31 (0.58)	.19	-1.36 (0.58)	.18
Psychiatric course trajectory								
Healthy controls	Ref		Ref				Ref	
Remission	10.30 (0.82)	<.001	-3.12 (1.12)	.002			0.63 (0.84)	.53
Recurrence	7.82 (1.66)	<.001	-0.01 (1.70)	.99			0.42 (1.23)	.67
Chronic course	14.65 (1.32)	<.001	3.65 (1.48)	<.001			0.94 (1.08)	.35
Symptom dimensions								
Anxiety arousal	24.28 (0.04)	<.001			12.49 (0.06)	<.001	12.51 (0.06)	<.001
Anxiety arousal x time	1.49 (0.02)	.14			0.90 (0.03)	.37	0.86 (0.03)	.39
Avoidance behaviour	23.28 (0.02)	<.001			10.58 (0.03)	<.001	10.57 (0.03)	<.001
Avoidance behaviour x time	-1.40 (0.01)	.16			0.17 (0.01)	.87	0.14 (0.01)	.89

^a^Analyses corrected for covariates age, sex, education, partner status, number of somatic illnesses and comorbid depression

Model 1: baseline psychiatric status and course trajectories; Model 2: baseline psychiatric status and symptom dimensions; Model 3: final model

In Model 1 the degree was examined to which course trajectories contribute to long-term disability across anxiety disorders. Baseline psychiatric status and chronic course remained significant predictors. Also SAD x time, PDA x time, GAD x time and multiple AD x time remained significant. This indicates that during follow-up the impact of baseline SAD, PDA, GAD and multiple AD on long-term disability became smaller.

In Model 2 baseline psychiatric status and symptom dimensions were assessed together. The results show that all coefficients decreased notably and only SAD, GAD, multiple AD, anxiety arousal and avoidance behaviour remained significant. PDA and PD did not remain significant. This indicates that anxiety arousal and avoidance behaviour lead to more long-term disability in anxiety disorders, which especially explain the found disability levels in PD and PDA.

In Model 3, baseline psychiatric status, course trajectories and symptom dimensions were assessed together. SAD, GAD, multiple AD, anxiety arousal and avoidance behaviour remained significant but course trajectories not. Furthermore, SAD x time and GAD x time remained significant. This indicates that the impact of SAD and GAD on long-term disability remained irrespective of course and symptom dimensions.

## Discussion

The results showed that all anxiety disorders were associated with more disability over 4 years of time compared to healthy controls, though differences became smaller over time.

Long-term disability was most prevalent in participants with SAD and multiple AD, and lowest in PDA and PD. GAD had an intermediate position. Furthermore, we found differences between course trajectories; a chronic course predicts long-term disability better than remission and recurrence. However, symptom dimensions seem to be stronger predictors of long-term disability than course trajectories.

Our finding that long-term disability is more prevalent in SAD and multiple AD compared to other anxiety disorders, is in line with previous results [36, 37] Because SAD and multiple AD are more associated with a chronic course than other anxiety disorders [20, 22, 23] and a chronic course is associated with more disability, [21] we expected that long-term disability is more prevalent in SAD and multiple AD. Our results showed that a chronic course is indeed associated with long-term disability. Avoidance behaviour was also associated with long-term disability in our study. We hypothesised that long-term disability would be high in SAD, PDA and multiple AD because of the high levels of avoidance behaviour in these disorder. However, PDA had a low position. So the assumption that avoidance behaviour predicts long-term disability better than anxiety arousal seems to be debatable.

Our findings showed intermediate associations for long-term disability and GAD. The association between GAD and disability may be particularly strong because worry is pervasive and can be focused on any area (more general concerns, including social situations and physical concerns), whereas the focus of distress for SAD, PDA and PD is more narrow. Nevertheless, this finding is only partly in line with previous results. Naragon-Gainey et al. [12] found that GAD at baseline was more associated with severe disability in certain areas (i.e. work, household, family, private leisure) compared to SAD and PDA/PD. However, after 2 years participants with GAD at baseline experienced no more disability than participants with SAD and PDA/PD. Previous research showed that comorbidity between GAD and other mental disorders is high (e.g. major depressive disorder) [5, 38–41]. Possibly, this high comorbidity can explain the inconsistent results among previous research. However, other research pointed out that the disability seen in GAD cannot be explained by comorbidity [42].

The results showed that long-term disability is lowest in PDA and PD which is partly in line with other studies [12, 13]. As mentioned in our previous study [11], disability in PDA and PD is generally associated with physical disability [14, 37]. Unfortunately, physical disability is not measured by the WHODAS-II very well and we can therefore not establish whether PDA and PD participants in this study have more physical disability compared to other participants.

Baseline psychiatric status and symptom dimensions were stronger predictors for long-term disability than course trajectories. This indicates that participants with SAD, GAD and multiple AD remain more disabled than PDA and PD, despite the course. Furthermore, although remission rates were relatively high, participants with anxiety disorders at baseline remain more disabled than healthy controls after 4 years. Possibly, baseline psychiatric status contains important information which was not measured in our study, such as age of onset, environmental factors, personality characteristics, and premorbid functioning. Another possibility is that when participants remit over 4 years, subthreshold anxiety symptoms may still be present and cause disability. Previous research indeed showed that subthreshold anxiety is also associated with long-term disability [43, 44].

This study has several strengths. Adequate analyses were performed because of the structured psychiatric interview, the large representative sample, and the longitudinal design. LMM analyses made it possible that all available information was used, even from participants with partly missing data. This study has also limitations. First, not all anxiety disorders are included in NESDA (e.g., obsessive compulsive disorder, posttraumatic stress disorder and specific phobia). However, in this study DSM-IV diagnoses were used instead of DSM-5. In DSM-5 obsessive compulsive disorder and posttraumatic stress disorder do not belong to the anxiety disorders chapter anymore and are discussed in separate chapters. Next, participants with depressive disorders were not excluded but analyses were corrected for comorbid depression. Furthermore, there can be differences in disability in the duration of a chronic disorder. For example, having a chronic anxiety disorder for 20 years causes possibly a different level of disability than having a chronic anxiety disorder for 5 years.

## Conclusions

Generally, long-term disability was highest in participants with SAD and multiple AD, followed by participants with GAD, and lowest in participants with PDA and PD. This means that various anxiety disorders have different disability levels over 4 years of time, so diagnostic distinction is important for treatment focus. When there is more than one anxiety disorder present there must be examined which anxiety symptoms are the most severe and disabling for the patient so treatment can focus on these symptoms. Our results show that anxiety arousal and avoidance behaviour lead to more long-term disability in anxiety disorders compared to course trajectories. Taken together, symptom dimensions in anxiety disorders give important information about disability over time. The use of symptom dimensions could eventually give more insight in all the complex associations in psychopathology and determine how psychiatric problems develop over time. The efficacy of transdiagnostic interventions for anxiety disorder patients (e.g. acceptance and commitment therapy and cognitive behavioural therapy) which are targeting these underlying processes might be evaluated or new interventions can be developed in order to prevent long-term disability. The results of our studies support the concepts of the DSM-5, which includes dimensional aspects of diagnosis along with categories.
